# Respiratory viruses interacting with cells: the importance of electrostatics

**DOI:** 10.3389/fmicb.2023.1169547

**Published:** 2023-06-27

**Authors:** Daniel Lauster, Klaus Osterrieder, Rainer Haag, Matthias Ballauff, Andreas Herrmann

**Affiliations:** ^1^Institut für Pharmazie, Biopharmazeutika, Freie Universität Berlin, Berlin, Germany; ^2^Institut für Virologie, Freie Universität Berlin, Berlin, Germany; ^3^Institut für Chemie und Biochemie, SupraFAB, Freie Universität Berlin, Berlin, Germany

**Keywords:** SARS-CoV-2, variants, electrostatic interaction, spike protein, surface charge

## Abstract

The COVID-19 pandemic has rekindled interest in the molecular mechanisms involved in the early steps of infection of cells by viruses. Compared to SARS-CoV-1 which only caused a relatively small albeit deadly outbreak, SARS-CoV-2 has led to fulminant spread and a full-scale pandemic characterized by efficient virus transmission worldwide within a very short time. Moreover, the mutations the virus acquired over the many months of virus transmission, particularly those seen in the Omicron variant, have turned out to result in an even more transmissible virus. Here, we focus on the early events of virus infection of cells. We review evidence that the first decisive step in this process is the electrostatic interaction of the spike protein with heparan sulfate chains present on the surface of target cells: Patches of cationic amino acids located on the surface of the spike protein can interact intimately with the negatively charged heparan sulfate chains, which results in the binding of the virion to the cell surface. In a second step, the specific interaction of the receptor binding domain (RBD) within the spike with the angiotensin-converting enzyme 2 (ACE2) receptor leads to the uptake of bound virions into the cell. We show that these events can be expressed as a semi-quantitative model by calculating the surface potential of different spike proteins using the Adaptive Poison-Boltzmann-Solver (APBS). This software allows visualization of the positive surface potential caused by the cationic patches, which increased markedly from the original Wuhan strain of SARS-CoV-2 to the Omicron variant. The surface potential thus enhanced leads to a much stronger binding of the Omicron variant as compared to the original wild-type virus. At the same time, data taken from the literature demonstrate that the interaction of the RBD of the spike protein with the ACE2 receptor remains constant within the limits of error. Finally, we briefly digress to other viruses and show the usefulness of these electrostatic processes and calculations for cell-virus interactions more generally.

## 1. Introduction

Electrostatic interaction of proteins with highly charged natural polyelectrolytes such as DNA is a long-standing problem of biophysics (Record et al., 1976, 1978) and is known to play a major role in many biological processes (Achazi et al., 2021). Glycosaminoglycans (GAGs) present a class of important natural polyelectrolytes that can interact with a great variety of proteins in a well-defined manner (Toledo et al., 2021; Ricard-Blum and Perez, 2022; Vallet et al., 2022). In particular, highly charged GAGs such as heparan sulfate (HS) play a central role in the organization of the extracellular matrix (Karamanos et al., 2021, 2022). Here, heparan sulfate proteoglycans (HSPG) consist of transmembrane, glycosyl-phosphatidyl-inositol-anchored, or secreted proteins onto which the highly negatively charged HS chains are attached (Condomitti and de Wit, 2018). HSPG can bind many different proteins including extracellular matrix proteins, growth factors, morphogens, cytokines and chemokines (Karamanos et al., 2021). Moreover, HSPG act as attachment factors for a number of viruses and bacteria (Chang et al., 2011; Cagno et al., 2019). Interacting with HSPG increases the concentration of virions on the cell surface, thus enhancing their chances for binding their cognate receptor(s) for cell entry. The length and pattern of sulfation are tissue- and cell type-specific (Marques et al., 2021), and, therefore, likely play an important role in pathogen tropism (Lee et al., 2022).

**Figure 1:**
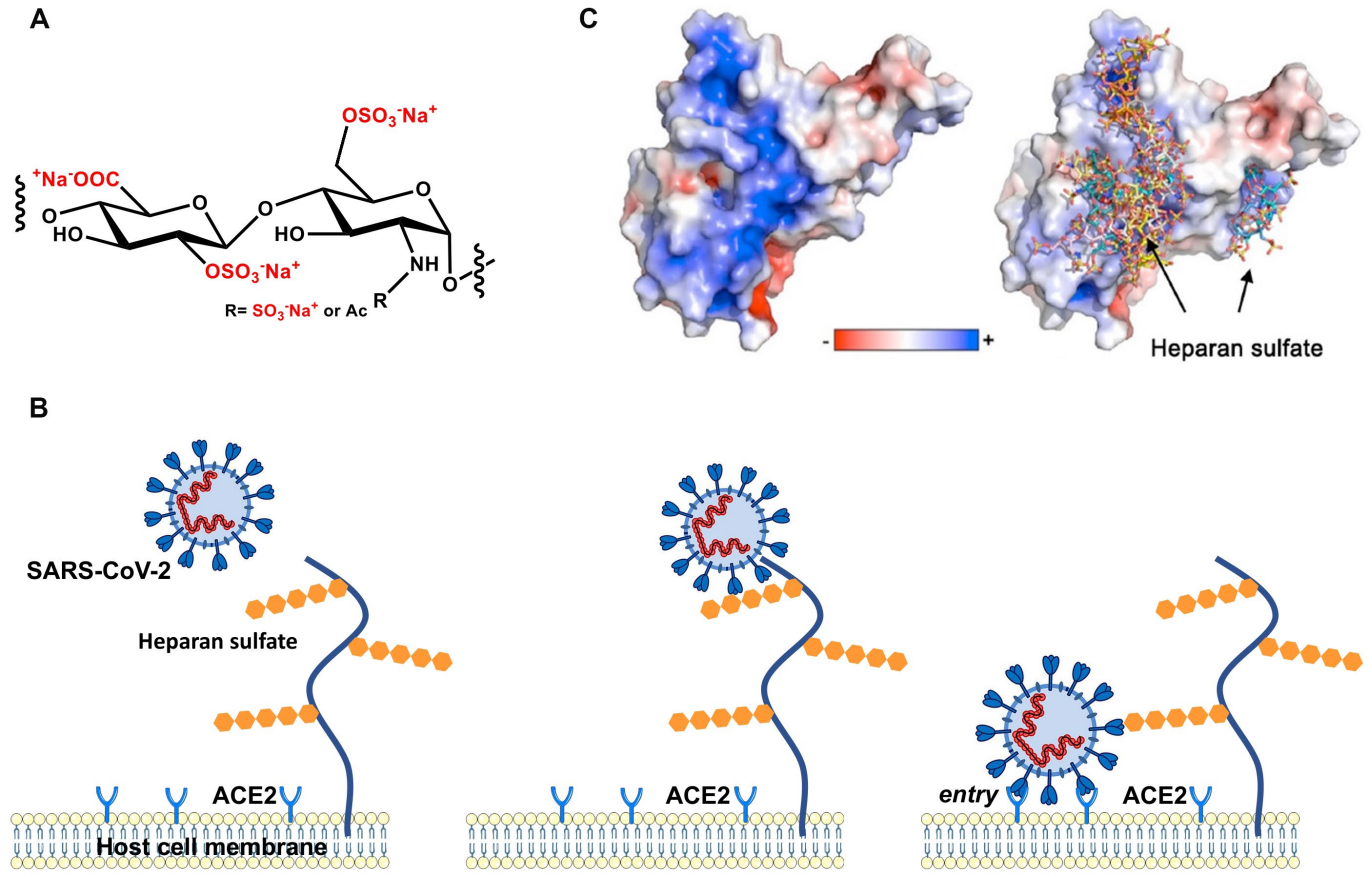
Early stages of cellular infection by SARS-CoV-2. **(A)** Repeating unit of heparan sulfate. **(B)** In the first step, S interacts closely with heparan sulfate attached to HSPG by strong electrostatic interactions. In a second step, interaction of S with the ACE2 receptor initiates uptake of the virion into the cell by endocytosis and, ultimately, S-mediated fusion of the virus envelope with endosomal membranes. **(C)** The electrostatic potential map of the receptor binding domain (RBD) of the wild-type (Wuhan) spike glycoprotein is represented. Positively charged amino acids located on the surface of the S homotrimer interact strongly with the highly negatively charged heparan sulfate moieties of the HSPG.

With the advent of the pandemic caused by severe acute respiratory syndrome coronavirus-2 (SARS-CoV-2), virus entry into cells has become a pressing and much-studied topic. The viral homotrimeric spike (S) glycoprotein mediates binding and subsequent steps of the early phase of host cell infection. Recent work has clearly demonstrated that the interaction of SARS-CoV-2 spike with HSPG on the surface of the target cell is the first and critical step in the infection process (Clausen et al., 2020; Kim et al., 2020; Liu et al., 2021; Nie et al., 2021; Kearns et al., 2022). In Figure 1, we show this process schematically: A patch of positive charge on the spike protein interacts closely with the highly negative charges of the HS chains of the HSPG. In a second step, the virion can find and interact with its specific host cell receptor for cell entry. Typically, angiotensin-converting enzyme 2 (ACE2) serves as a cellular receptor, but other cell surface molecules such as neuropilin-1 (Daly et al., 2020) and integrin (Liu J. et al., 2022) have also been shown to serve in that role. Hence, the strong electrostatic interaction of a highly charged GAG with the envelope proteins of SARS-CoV-2 is central for this critical first step of infection. This fact is supported by the observation that highly-charged synthetic polyelectrolytes (Nie et al., 2021) or heparin (Mycroft-West et al., 2020) can inhibit virus infection by competing with HSPG (Hoffmann et al., 2022). The electrostatic interaction between virus and HS may become even more important when considering the mutations of the S protein and the much higher infectivity of SARS-CoV-2 variants. As revealed by molecular modeling (Jawad et al., 2022; Nie et al., 2022; Pascarella et al., 2022; Fantini et al., 2023a) when compared with the authentic Wuhan-type spike, the positive patch on the surface is enlarged in its counterparts of the Delta and the Omicron variants, which suggests a much stronger binding of the virion to HSPG (see below). This finding underscores the central role of electrostatic interaction for virus infection in the early phase of binding to host cells (Cagno et al., 2019).

Studies on the interaction of the SARS-CoV-2 virion with HSPG have renewed the general interest in complex formations of GAGs with proteins. It has been posited that the positive patches on the surface of proteins can be rationalized in terms of the Cardin-Weintraub sequences (Cardin and Weintraub, 1989; Cardin et al., 1991; Rudd et al., 2017), where two or three basic amino acids are grouped together with hydrophobic amino acids. Recently, Kim et al. have traced back the enhanced binding of the SARS-CoV-2 virion to HSPG compared to SARS-CoV-1 to additional Cardin-Weintraub sequences on its spike protein (Kim et al., 2020). Moreover, Liu et al. have found that the binding of well-defined HS oligomers to the receptor binding domain (RBD) depends on their length and sequence. Specifically, a minimum length of 6 repeating HS units was found to be necessary for binding (Liu et al., 2021).

As mentioned above, the general theory of the interaction of polyelectrolytes with proteins has been worked out many years ago (Record et al., 1978), and has since been applied to many natural and synthetic polyelectrolytes (Xu et al., 2019; Achazi et al., 2021). The theory is based on Manning’s prediction of counterion condensation (Manning, 1969) of polyelectrolyte chains: A part of the counterions of a highly negatively charged polyelectrolyte will be firmly immobilized or condensed on the chain. Interaction with a patch of positive charge on a protein will liberate a corresponding number of these condensed counterions. The counterion release will then increase the entropy of the system, providing a strong driving force for complex formation of the polyelectrolyte and the protein. Since the condensed counterions are firmly bound to the polyelectrolyte, they will act much in a way of a chemically-bound species during complex formation. Hence, their activity enters directly in the mass action law and the free energy of binding scales logarithmically with the salt concentration in the system (Record et al., 1978). Strong interaction of a polyelectrolyte with a protein has thus two ingredients: (i) a patch of positive amino acids of sufficient size, that is, of typically 3 to 4 cationic amino acids grouped in a Cardin-Weintraub sequence, and (ii) a highly-charged polyelectrolyte chain at which counterion condensation takes place.

This model has met with gratifying success when applied to the binding of DNA to various proteins (Lohman and Mascotti, 1992; Mascotti and Lohman, 1993; Bergqvist et al., 2001, 2003; Achazi et al., 2021) or to the interaction of synthetic polyelectrolytes with proteins (Xu et al., 2019). Also, the interaction of heparin with various proteins can be rationalized in terms of this model (Olson et al., 1991; Thompson et al., 1994; Mascotti and Lohman, 1995; Hileman et al., 1998; Friedrich et al., 2001; Capila and Linhardt, 2002; Schedin-Weiss et al., 2002a,b, 2004; Jairajpuri et al., 2003; Seyrek and Dubin, 2010; Walkowiak et al., 2020; Malicka et al., 2022). Taken together, all the cited studies demonstrate clearly that the counterion release model provides a valid and fully quantitative description of complex formation between polyelectrolytes and proteins.

Here, we review and discuss recent findings on virus attachment to HSPG and cell surfaces and compare our results with the counterion release model of complex formation between highly charged polyelectrolytes and proteins. Special emphasis is put on the results obtained with SARS-CoV-2. We show that the results provide a firm basis for discussing the role of electrostatic interaction for virus infection in general. The paper is organized as follows: Section “Electrostatic interaction of proteins with polyelectrolytes” contains a brief survey of the modeling of electrostatic interaction. In section “Electrostatics in virus infection”, the model will be used for a comparison with experimental results obtained with SARS-CoV-2 and related viruses. The extension of these ideas to less-well studied viruses is given in section “Human Respiratory Syncytial Virus and human Metapneumovirus”, and a brief conclusion will wrap up the discussion.

## 2. Electrostatic interaction of proteins with polyelectrolytes

### 2.1. Proteins interact with polyelectrolytes by counterion release even at high ionic strength

**Figure 2:**
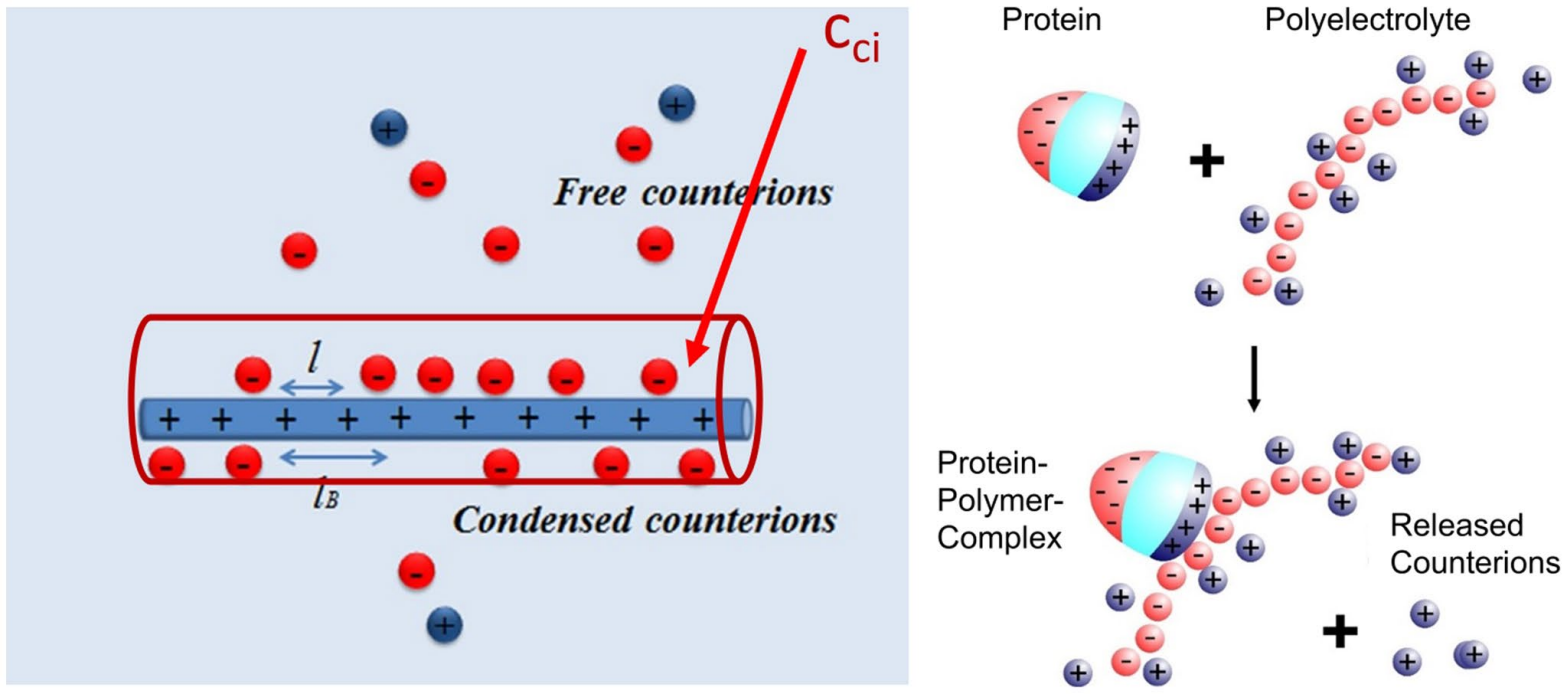
Counterion release as main driving force for complex formation between a polyelectrolyte and a protein (Achazi et al., 2021). Left-hand side: A part of the counterions of the highly charged macroion is condensed, that is, highly correlated to the macroion and does not contribute to the measured osmotic pressure. This enrichment of ions near the macroion can be characterized by a surface concentration c_ci_ which for heparin is of the order of 1 M (Walkowiak and Ballauff, 2021; Malicka et al., 2022). Right-hand side: Complex formation of a protein with such a macroion is due to the close interaction of a patch of positive charge on the surface of the protein with the polyelectrolyte. The positive patch becomes a multivalent counterion of the macroion thus releasing a concomitant number of monovalent counterions into the bulk solution. The free energy of binding hence consists of an entropic term due to this effect and a term due to interaction at direct contact [*cf.* the discussion of eq. (2) below].

As already discussed in previous expositions of the subject, the main driving force for the binding of highly charged polyelectrolytes to proteins is the release of counterions. Figure 2 shows this process in a schematic fashion: A fraction of the counterions is condensed onto the polyelectrolyte. The criterion for counterion condensation is the charge parameter(Manning, 1969) *ξ = λ_b_/l*, where *l* is the distance of the charges along the chain. λ_b_ is the Bjerrum-length, the distance between two elementary charges at which electrostatic interaction is in the order of the thermal energy *k_B_T*. λ_b_ is 0.7 nm in water at 25°C. If *ξ >* 1, a fraction 1–1/*ξ* of the counterions will be condensed, that is, it will be strongly correlated to the macroion [“Manning condensation” (Manning, 1969)]. As an important consequence, condensed counterions will not contribute to the osmotic pressure of the system. Heparin is among the natural polyelectrolytes with the highest charge and characterized by a charge parameter *ξ =* 2.84 (Minsky et al., 2013; Walkowiak et al., 2020). If such a highly charged polyelectrolyte forms a complex with a protein, a patch of positively charged amino acids on the surface of the protein becomes a multivalent counterion of the polyelectrolyte, thus releasing a corresponding number of counterions. The increase of entropy effected by this counterion release is a major driving force for complex formation. Counterion condensation can also be characterized by the surface concentration *c_ci_*, which can be estimated from the number of condensed ions per unit length and a diameter of the cylinder in which the ions are confined (see Figure 2; Manning, 1978; Xu et al., 2017, 2018, 2019). Estimates of this concentration are in the order of 1 M for typical, highly charged polyelectrolytes such as DNA or heparin (Xu et al., 2019). This concentration is much higher than the physiological salt concentration of 0.15 M and a release of condensed ions to the bulk phase will therefore result in a considerable gain of free energy of nearly 2 k_B_T per ion.

The positive patch on the protein must have a certain size of 2 or 3 cationic amino acids to act in this way. This fact is the background for the finding that Cardin-Weintraub sequences (Cardin and Weintraub, 1989; Cardin et al., 1991) in which cationic amino acids B are grouped together with hydrophobic moieties X as “XBBXBX” and “XBBBXXBX” sequences/motifs. Rudd et al. (2017) have reanalyzed these GAG-binding sites for a large number of systems and concluded that sequences act only through their presence on the protein surface, exactly as shown in Figure 2.

These considerations can be put into a quantitative frame as discussed recently (Xu et al., 2019; Achazi et al., 2021; Walkowiak and Ballauff, 2021; Malicka et al., 2022): the free energy of binding $\Delta G_{b}\left(T,c_{s}\right)$ follows from the binding constant *K_b_* through


(1) $$\Delta G_{b}\left(T,c_{s}\right)=-\mathrm{RTln}\;K_{b}$$

which characterizes the equilibrium between the components and the complex of the polyelectrolyte with the protein. The condensed counterions liberated during complex formation will act in this equilibrium as a chemical component, and the free energy of binding will thus scale with ln *c_s_* where *c_s_* is the salt concentration in the system (Record et al., 1978). The free energy derived from this model is given in eq. (2) (Achazi et al., 2021).


(2) $$\Delta G_{b}\left(T,c_{s}\right)=\mathrm{RT}\Delta n_{ci}\ln c_{s}-\mathrm{RT}\frac{2}{55.6}\Delta wc_{s}+\Delta G_{res}\;$$

The first term is due to the effect of counterion release as described above scaling with the logarithm of the salt concentration in the solution. The term $\Delta n_{ci}$ is the number of released counterions. The second term addresses the change of hydration during complex formation. Here *Δw* is the net contribution to the free energy of binding. In many cases studied so far, this term is small and can be disregarded in a first approximation. Finally, the third term $\Delta G_{res}$ is the free energy resulting from the interaction at direct contact, mainly by salt bridges and hydrogen bonding (Xu and Ballauff, 2019; Walkowiak and Ballauff, 2021). We note that any distinct pattern of the arrangement of charged groups of polyelectrolytes is not considered by this theoretical approach (see also below “Effects of finite length of the polyelectrolyte: Heparin/HS must exceed a certain length to bind to proteins”).

Eq. (2) has been applied repeatedly to the analysis of heparin interacting with different proteins [*cf.* the discussion in Achazi et al. (2021)]. In all cases studied so far, it was found that *Δw* = 0; it follows that plots of ln *K_b_* against ln *c_s_* are strictly linear. Extrapolation to a salt concentration of 1 M was used to obtain $\Delta G_{res}$ , whereas the number of released counterions $\Delta n_{ci}$ was found to be typically around 3. A more recent analysis of the binding of lysozyme to heparin corroborated the main conclusions of earlier work (Malicka et al., 2022). The second term in eq. (2) describing the effect of hydration was analyzed in detail. It was found that hydration as embodied in *Δw* hardly contribute to the measured free energy of binding $\Delta G_{b}$ for salt ions located approximately in the middle of the Hofmeister series (Malicka et al., 2022). Hence, for a first approximation of the binding constant, it suffices to take into account only the first and the third term of eq. (2).

### 2.2. Effects of finite length of the polyelectrolyte: heparin/HS must exceed a certain length to bind to proteins

The above theoretical model of counterion condensation assumes a rodlike polyelectrolyte of infinite length. If the macroion has a finite length, however, the fraction of condensed counterions decreases and vanishes at a critical length. This problem has been considered first for DNA by Record et al. (1978) who showed that the extent of counterion on a polyelectrolyte must be corrected by a term that scales with 1/*N*, where *N* is the degree of polymerization. Netz (2003) and Kim and Netz (2015) reconsidered the problem for a rod-like polyelectrolyte modeled as a cylinder of finite radius. The average degree of counterion condensation was given in an analytical expression for salt-free solutions, while Manning developed an expression that describes effects at the termini of rod-like polyelectrolytes (Manning, 2008). Minsky et al. (2013) demonstrated the effect of finite length in a careful study of the electrophoretic mobility of heparin oligomers at low ionic strength. By measuring the effective charge of oligomers of different average chain lengths, the authors could show that the average number of condensed counterions increases with increasing chain lengths.

One can conclude from the studies that counterion condensation is greatly diminished for short macroions and that counterion release is no longer effective for the binding of polyelectrolytes to proteins. This fact results in a much weaker interaction of heparin oligomers with various proteins. Thus, Hernaiz and colleagues found the dissociation constant *K_D_* of a complex between a synthetic peptide form the human amyloid peptide P with heparin increases by one order of magnitude when going from the polymer heparin to the tetrasaccharide (Hernaiz et al., 2002). Similar findings have since been reported by other groups (Zhang et al., 2015; Nguyen and Rabenstein, 2016; Kohling et al., 2019). Recently, Liu et al. demonstrated this effect in a carefully study assessing the interaction of GAGs of various structures and lengths with the RDB of SARS-CoV-2 (Liu et al., 2021). They have used a library of well-defined HS oligosaccharides to determine the conditions that must be met for a HS oligosaccharide to bind to the S proteins of wt SARS-CoV-2. They found that the binding of HS depends on both the length and the pattern of the arrangement of the sulfate groups. In addition to a minimum length equal to that of hexamers, at least 8 sulfate groups were required for significant binding. Octasaccharides with 9 or 12 sulfate groups showed much stronger binding. Polymers composed of trisulfated repeating units displayed the highest affinity implicating that a high charge density favors binding. These conditions for binding HS were found for both the RBD and the S protein of wt SARS-CoV-2, with the affinity for the S protein being about an order of magnitude higher. The latter was attributed to a further HS binding site at the furin-cleavage site in addition to a binding site at the RBD (Liu et al., 2021).

An important observation by Liu et al. was that a removal of only one sulfate from a hexasaccharide (see comparison of structure 90 or 91 of Liu et al.) caused a significant reduction in HS affinity to the full length S protein and – but to a lower extent – to the isolated RBD. The authors concluded that this result points to specific interactions of sulfates with the protein which would be in line with the HS sulfate code hypothesis. The latter suggests that specific HS epitopes on the cell surface may allow to recruit specific HS-binding proteins (Xu and Esko, 2014). However, Liu et al. did not preclude that electrostatic interactions are of relevance for HS binding to the S protein.

Nie et al. (2021) also found, using linear polyglycerol sulfates (LPGS), that short-chain LPGS with a low number of sulfate groups (6 repeating units) had no inhibitory effect on the infection of Vero E6 cells by wt SARS-CoV-2, but long-chain LPGS with 20 repeating units caused a strong inhibition of infection. Increasing the degree of sulfation led to a greater reduction of infected cells, confirming that a high charge density given by sulfate groups promotes the inhibitory effect of LPGS. An independent support of this conclusion was provided by Hao et al. using a heparan sulfate microarray. They have shown for both the full-length S protein and the RBD of SARS-CoV-2 that the stepwise addition of 6-O-sulfate groups gradually enhanced binding. At this stage of investigation, the authors concluded that the number of sulfate groups is relevant for binding, but not the length of the HS (Hao et al., 2021). These findings are in accord with the theory of electrostatic interaction as described above: Counterion condensation is the necessary condition for electrostatic interaction of sufficient strength and only operative for chains exceeding a minimum chain length. However, so far, the theory does not consider specific arrangements of the ligands for binding (Xu and Esko, 2014). Further studies are warranted if and how such specific arrangements can be integrated into theoretical considerations.

## 3. Electrostatics in virus infection

The role of virion binding to HSPG as the first step of infection has been the subject of a careful review by Cagno et al. (2019). The authors showed that this first interaction is clearly the decisive step for the efficiency of infection of many viruses. Here, we will analyze in detail the relevance of opposite charges for the initial interaction between virus and target cell focusing on coronaviruses.

### 3.1. Wildtype SARS-CoV-2

As all coronaviruses, SARS-CoV-2 uses its homotrimeric envelope spike glycoprotein (S) to attach to the host cell. The wild-type (Wuhan) S features 1,273 amino acid residues in total, and 1,208 of them form the ectodomain. Each monomer consists of two subunits, S1 and S2 which are a result of proteolytic cleavage. S2 contains the hydrophobic transmembrane domain anchoring the protein to the envelope. Cleavage of S into the two subunits is performed by host cell enzymes that include furin and TMPRSS2 at distinct sites, and the process is essential for the subsequent conformational change of the protein that exposes the hydrophobic peptides triggering fusion (Hoffmann et al., 2020). Taking advantage of the knowledge of SARS-CoV-1 which caused a small-scale pandemic in 2002/2003, ACE2 was quickly identified as a specific receptor for SARS-CoV-2 (Hoffmann et al., 2020). S1 binds to ACE2 with a specific receptor-binding motive (RBM) localized in the RBD, which covers residues 333 to 527 (residue numbers given correspond to the original Wuhan S sequence).

However, it is not the interaction of the spike protein with ACE2 which is decisive for initial binding but the electrostatic interaction of the spike protein with the HSPG. Evidence for the electrostatic nature of the first step of virus binding to cell surfaces also comes from inhibition studies (Mycroft-West et al., 2020; Nie et al., 2021; Tree et al., 2021; Guimond et al., 2022). Highly charged polyelectrolytes such as heparin or the synthetic linear polyglycerol sulfate (Nie et al., 2021) tightly interact with the RBD electrostatically so that binding of the virion to HSPG is no longer possible. MD simulations provided additional support for this interaction directly and revealed that the more flexible linear polyglycerol sulfates could bind more tightly to the RBD exceeding the entropic costs by far, ultimately leading to a stronger inhibition (Nie et al., 2021).

To gain detailed insight into the interaction between S and anionic ligands based on opposite charges, the analysis of the surface potential of the protein and the possible identification of potential binding sites is extremely helpful. Here, we demonstrate this fact by calculating and visualizing the surface potential of the ectodomain or selected motifs of S using the software “Adaptive Poison-Boltzmann-Solver” (APBS; Jurrus et al., 2018). This program allows us to calculate numerically the surface potential of a given protein and visualize the result.

**Figure 3:**
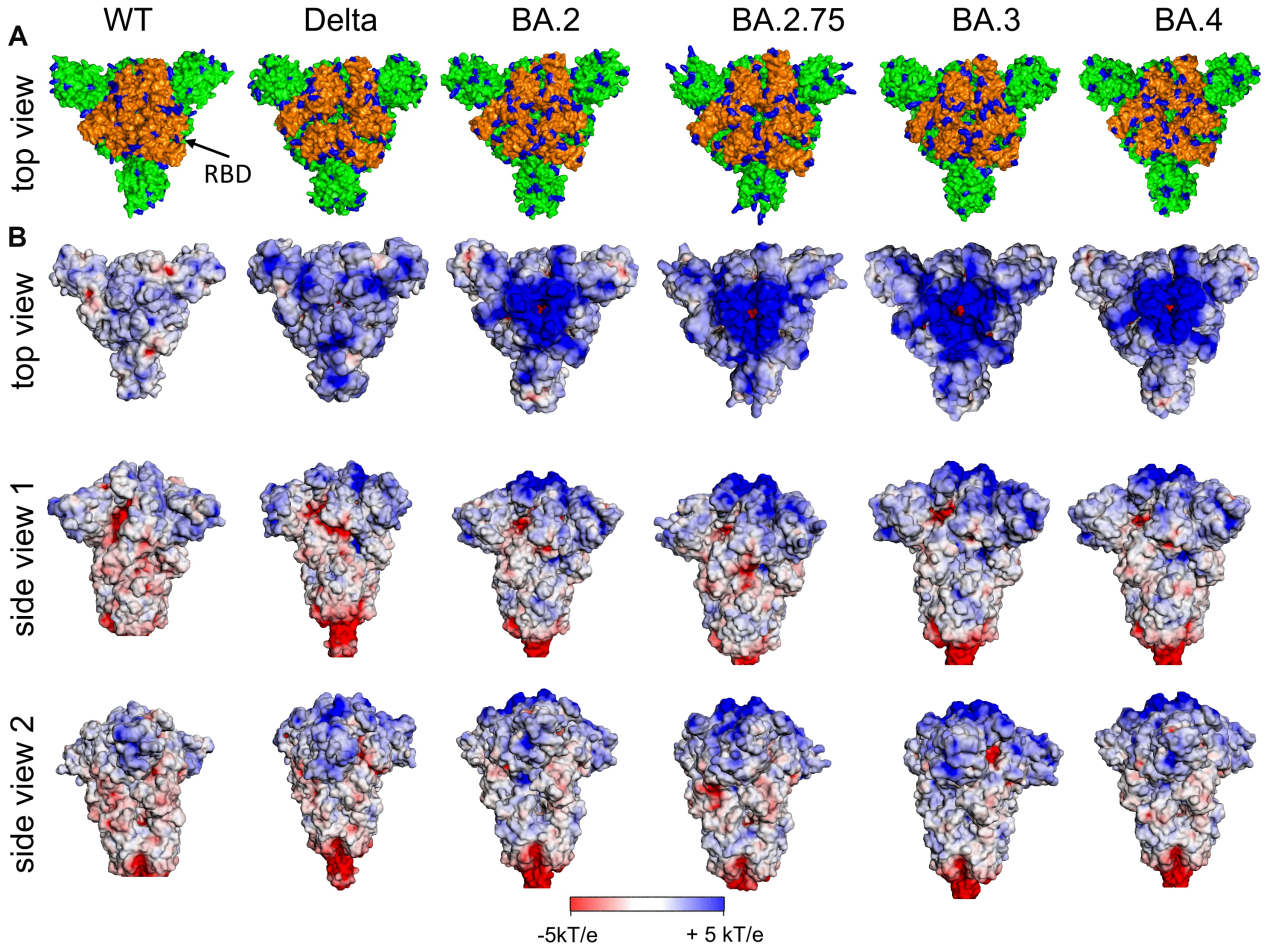
**(A)** Distribution of positively charged amino acids Arg and Lys (blue) in the top domain [RBD orange (arrow)] and **(B)** surface potential of the (Wuhan, wt) SARS-CoV-2 S ectodomain and various variants of concern (VoC) in the closed state of RBDs at pH 7.0 [pdb: 7QUS (wt); 7SBK (Delta); 7XIX (BA.2); 7YQU (BA.2.75); 7XIY (BA.3); 7XNS (BA.4)]. Note, in the interest of stability the furin cleavage site in the stem region has been removed for the various spike proteins as usually done for 3D structure determination and other experimental setups.

In Figure 3 (left), the distribution of positively charged amino acids in the top region and the surface potential of the ectodomain of the S protein of the original Wuhan (wt) SARS-CoV-2 are shown. It is obvious that the most distal part of the ectodomain, i.e., the top region, is characterized by a positive surface potential, while the stem region is characterized by domains with a neutral or even negative potential. Thus, the top region provides a potential target for negatively charged ligands.

**Figure 4:**
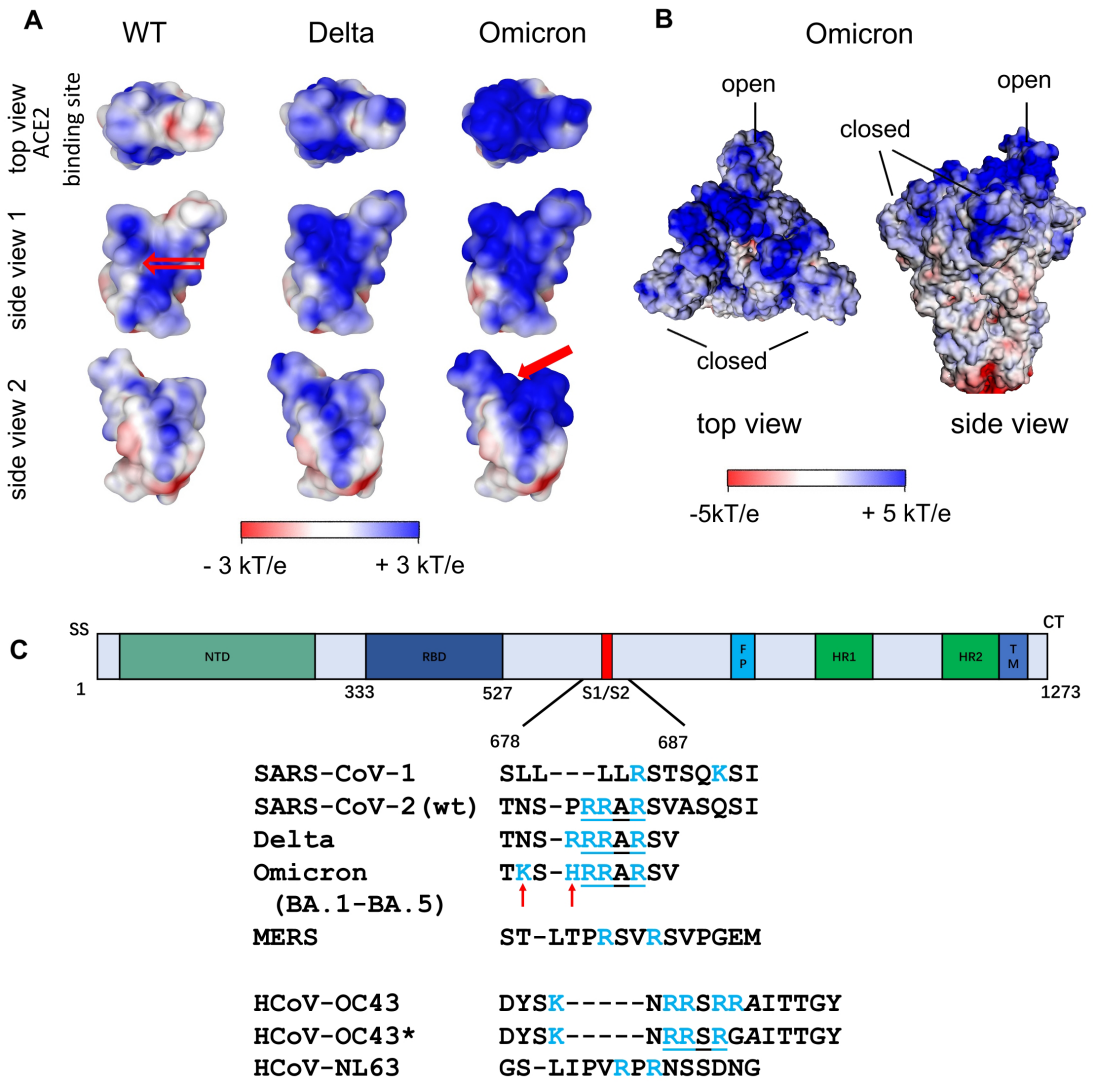
**(A)** Surface potential map of the RBD of the Wuhan (wt), the Delta and the Omicron variant at pH 7.0 (Nie et al., 2022). The top view corresponds to the top view of S with the open conformation of the RBD, i.e., the view of the ACE2 binding site. The stretch of positively charged amino acids in the Wuhan RBD (see empty arrow in “side view 1”) has been proposed as heparan sulfate binding site. A second binding site of similar affinity was found for Omicron variants (see filled red arrow in “side view 2”; Nie et al., 2022). **(B)** Surface potential of the ectodomain of the trimeric S of the Omicron variant (B.1.1.529) with two RBDs being in the closed state and one RBD in the open state (pdb 7TGW). **(C)** Additional Cardin-Weintraub sites at the furin cleavage site of S Wuhan (wt) SARS-CoV-2 S and SARS-CoV-2 VOCs. For comparison, the corresponding sequences of SARS-CoV-1, MERS and of the endemic coronaviruses HCoV-NL63 and HCoV-OC43/HCoV-OC43* are shown. In contrast to HCoV-OC43, HCoV-OC43* has undergone multiple passages in cultured cells (de Haan et al., 2008).

Figure 4A (left) shows the surface potential map of the RBD for the wild type (Nie et al., 2021). Here, a channel of positive charge is seen on the surface of the RBD into which the strongly negative HS chain fits well. The SARS-CoV-2 RBD of the Wuhan strain with the positively charged amino acids (Figure 3A, left) contributes significantly to the positive potential of the distal region of the S ectodomain and is shown from various perspectives in Figure 4. Apart from the RBD binding site for ACE2 (top view), the positive surface potential of the segments shown in the side views is of specific interest. In particular, the extended stretch of a positive surface potential (Figure 4 left, wt, red arrow in “side view 1”) has been implicated as binding site for heparan sulfate by molecular modeling (Clausen et al., 2020; Nie et al., 2022). The analysis of the structure of S has revealed a well-defined RBD binding site for HS (Clausen et al., 2020; Liu et al., 2021). Since S comes as a trimer, three binding sites for the HS chains are leading to a multivalent interaction with remarkable strength. Clausen et al. (2020) stabilized the “open” or “closed” RBD conformation by site-directed mutagenesis of S and found a comparable binding affinity of both states for HS (Clausen et al., 2020). It has been also shown that binding of HS to the RBD does not only support the transition of the RBD to the “open spike” conformation required for binding to ACE2, but also stabilizes this conformation (Clausen et al., 2020; Yin et al., 2022). Using cryo-EM, Clausen et al. found that a sulfated heparin-derived icosasaccharide fragment caused a significant increase in the total amount of bound ACE2 to the S-trimer of wt SARS-CoV-2, due to the increased proportion of S-trimers carrying one or two ACE2 and a concomitant decrease in unbound S-trimers. As Liu et al. (2021) has provided evidence that the binding affinity of ACE2 to the RBD is only marginally reduced in the presence of an RBD binding octasaccharide we surmise that HSPG could increase the probability of ACE2 binding to the RBD of the S protein by favoring the open conformation rather than enhancing the affinity of ACE2 to the RBD *per se*. Based on the observation that the affinity of ACE2 to the RBD is about 15 to 20 fold higher than that to the S protein (Wrapp et al., 2020) and on their own observations, Shang et al. (2020) suggested that the dynamics between open and closed conformation and a preference for the latter could explain the difference of ACE2 affinity between RBD and the S protein. Thus, enhancing just the fraction of S proteins being in the open conformation could already lead an increase of the amount of bound ACE2 without affecting the affinity of ACE2 to the RBD.

Based on studies of the role of specific N-linked glycans of S in supporting the conversion in the open conformation of the RBD and its stabilization, Kearns et al. (2022) have hypothesized in their review that HS can support or even replace the action of the specific cellular receptor (Puray-Chavez et al., 2021). For the S protomer, 22 N-linked glycosylation sites were predicted. Experimentally, it could be shown that at least 17 of them are glycosylated (Casalino et al., 2020; Walls et al., 2020; Watanabe et al., 2020). As with the spike proteins of other viruses, the extensive glycosylation of the protein surface masks the antigenic sites and thus provides a protective shield against the immune system which is supported by the high flexibility of the glycan structures. A subsequent study has shown both experimentally and by molecular modeling simulations that certain N-glycans–specifically the one linked to position N343–can facilitate the opening of the RBD and thus its binding to ACE2 (Sztain et al., 2021). Another molecular modeling study had concluded that an open conformation of the RBD can be stabilized by the glycans linked to N165 and N234 (Casalino et al., 2020). The N165 glycan stabilizes the up conformation by moving under the RBD when it has taken the up conformation (Harbison et al., 2022). The fact that the RBD binding site of HS significantly overlaps with the RBD binding site for N165 led Kearns et al. (2022) to hypothesize that HS can take over the function of the N165 glycan and thus promote the binding of RBD to ACE2. Puray-Chavez et al. (2021) has found that a lung adenocarcinoma cell line which did not express ACE2 can be infected by SARS-CoV-2 in the presence of HS. However, other cellular receptors such as neutrophilin-1 and integrin have been shown to mediate cell entry (Daly et al., 2020; Liu J. et al., 2022).

Additional strong support for the importance of electrostatic interaction comes from a comparison of SARS-CoV-1 and SARS-CoV-2. Kim et al. have called attention to a second cationic binding location at the furin cleavage site when comparing SARS-CoV-1 with SARS-CoV-2 S (Figure 4C; Kim et al., 2020). The new binding site which is preserved in the Delta and all currently dominating Omicron variants (BA.1-BA.5, XBB.1.5, XBB.1.16) features a sequence of amino acids that follows the classical Cardin-Weintraub scheme (alternating hydrophobic (X) and positively (B) charged amino acids as XBBXBX or XBBBXXBX; Cardin and Weintraub, 1989; Cardin et al., 1991), and presents an additional binding site for HS [*cf.* the discussion of this point by Liu et al. (2021)]. This new site should lead to a much stronger binding of SARS-CoV-2 S to HS when compared to that of SARS-CoV-1. Indeed, Kim et al. found that *K_D_* for the binding of the SARS-CoV-1 monomer to heparin is 0.5 μM, whereas the *K_D_* = 40 pM for the interaction in the case of SARS-CoV-2 S (Kim et al., 2020). This more than 10,000-fold increase of the binding strength can only be explained by the transition from a complex with a single binding site to a complex in which two HS chains are bound to S. A rough estimate can be done from previous work on the basis of eq. (2): $\Delta G_{res}$ was found to be 20 kJ/mol or 7.9 k_B_T for the interaction of lysozyme with heparin where $\Delta n_{ci}$ is 3 (Malicka et al., 2022). Similar values can be assumed for the interaction of the RBD with HS: Here, the patch on the surface is large enough so that 3 cationic amino acids can interact with HS. As discussed above, *Δw* can be set to zero in good approximation. Then, using eq. (2), we can estimate for a salt concentration of 0.15 M that *ΔG_b_* ≅ 7.9 + 5.7 = 13.6 k_B_T, which would result in a *K_D_* ≅ 1 μM. For SARS-CoV-1, which does not have this additional binding site at the furin cleavage site discussed above, Kim et al. found a value of *K_D_* = 0.51 μM, which is in the same order of magnitude (Kim et al., 2020). A second binding site appears at the furin cleavage site as shown for the wild type and the SARS-CoV-2 VOCs (see Figure 4). Now, the HS chains have two patches on the S monomer to which they can bind simultaneously. This means that the free energies of two binding sites increase dramatically, and the *K_D_* is predicted to be of the order of 1 pM whereas Kim et al. find a value of 40 pM from their surface plasmon resonance (SPR) experiments. Apart from the large variation of the *K_D_* measured by SPR between independent studies (see below), given the various stringent assumptions in this simple calculation of Kim et al. there is at least semi-quantitative agreement. Evidently, the enormous increase of the binding strength of S to heparin from SARS-CoV-1 to SARS-CoV-2 is in full accord with an additional binding site of HSPG to the S trimer (Figures 3, 4).

### 3.2. Higher cationic S surface charge of SARS-CoV-2 VoCs

Perhaps the most convincing evidence for the central role of electrostatics for the infection with SARS-CoV-2 comes from studies of its mutants. Several VoCs have turned out to be 2- to about 5-fold more infectious than the original Wuhan virus. This increased infectivity is caused by mutations primarily in the S ectodomain, which enhance binding affinity and/or support more efficient fusion with the target membrane (Cao et al., 2022a; Syed et al., 2022; Sun et al., 2023). These variants of increased fitness and/or resistance to vaccine protection/immune responses have displaced the original Wuhan virus, and then one VoC was replaced, sometimes in a matter of weeks, by the next more transmissible variant. The dominant VoCs in 2022 are Omicron strains, especially strains BA.4. and BA.5. The subvariants BA.2.75.2 and the Omicron BA.5 descendant BQ.1.1 are expected to become predominant in Western countries in the winter season 2022/2023 (Planas et al., 2022), while the Omicron variant XBB originated by recombination of two BA.2 descendants has become dominant in South and Southeast Asian countries (Tamura et al., 2022) and began to spread strongly in the U.S. in January 2023. These various Omicron species have more than 60 mutations with respect to the Wuhan virus, more than half of them in the ectodomain of the S monomer.

An important change along the successively evolving VoCs was the increase of positively charged amino acids in the S ectodomain, especially in the top region as shown in Figure 4 including the RBD (Figure 3A, orange). The additional positive amino acids on the surface led to an increased positive surface potential both in the closed and in the open state of the RBD (Figure 4B). The increase of positive charges is seen when analyzing subsequent variants leading to four more positive amino acids for the Delta variant and up to a total of nine more for the Omicron variants (Barroso da Silva et al., 2022; Nie et al., 2022; Pascarella et al., 2022; Fantini et al., 2023a,b). A second binding site for heparan sulfate in the RBD of Omicron variants is directly obvious from Figure 4A indicated by the filled red arrow in side view 2 (Nie et al., 2022). We hypothesize, that the marked increase of positive charges leads to a much stronger binding of HS to the S of Omicron variants.

**Figure 5:**
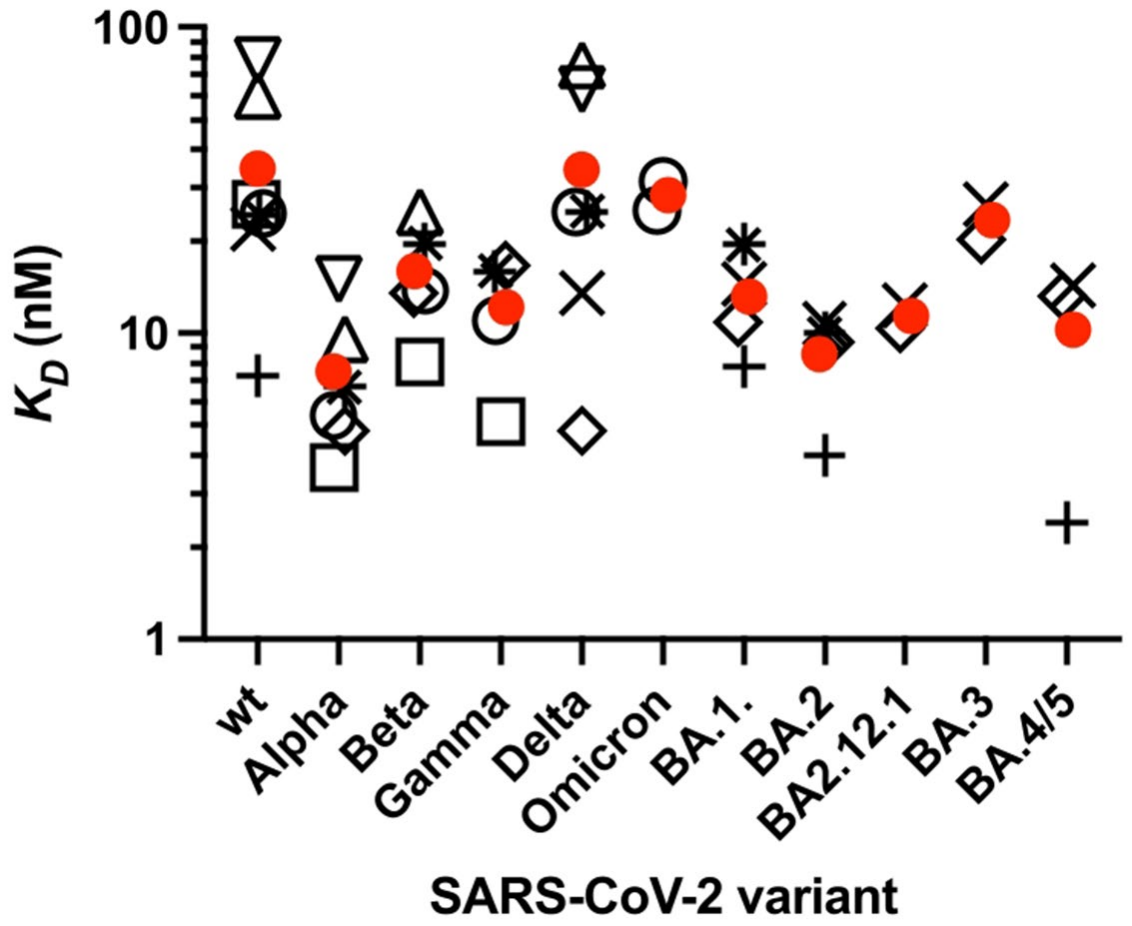
Affinity *K_D_* of the RBD of the spike protein of SARS-CoV-2 variants to ACE2 measured by SPR. *K_D_* was measured at 25°C or RT. Values are taken from several studies (open circle). Only studies that performed measurements on at least three SASR-CoV-2 variants were selected. Furthermore, only variants for which at least two, but typically three or more independent measurements were available are presented. Circles correspond to the isolated RBD, *K_D_* values of the RBD [open square (Han et al., 2021); open circles (Han et al., 2022); open triangle up (Cameroni et al., 2022); open triangle down (McCallum et al., 2021); plus sign (Nutalai et al., 2022; Tuekprakhon et al., 2022); (Dejnirattisai et al., 2022); × (Cao et al., 2022b); open rhombus (Cao et al., 2022a); star (Li et al., 2022)]. Average *K_D_* values are taken from each study. Mean values of these *K_D_* values are shown (red circles).

It is important to note that the interaction of ACE2 with the RBD is of similar strength for the different mutants (Han et al., 2022). A comparison performed by Han et al. recently revealed that the dissociation constant *K_D_* of the ACE2-S complex varies between 5 nM for the Alpha and 31 nM for the Omicron variant, whereas *K_D_* = 24.6 nM is found for the original Wuhan virus (Han et al., 2022). An overview of *K_D_* values of the interaction between ACE2 and the isolated RBD or the RBD of the complete ectodomain of the S-glycoprotein for different SARS-CoV-2 VoCs is shown in Figure 5. Only *K_D_* values measured by SPR are shown here, as this method has been most commonly used to characterize the affinity between ACE2 and the RBD. Preliminary data shows that the affinity of the recent variant XBB.1.5 is in the same order of the various Omicron variants (Yue et al., 2023). The *K_D_* values are distributed over a wider range and the data are afflicted by a rather large experimental error. No clear trend of decreasing *K_D_* values for the Omicron variants is evident within the present limits of error. Moreover, the free energy of the interaction between ACE2 and the RBD in units of k_B_T is given by the natural logarithm of *K_D_*, and differences between *K_D_* values as seen in Figure 5 will exert only a small influence on the complex formation between ACE2 and the RBD. Hence, the stability of the ACE2-S complex is comparable for all variants and cannot be responsible for their much higher infectivity as compared to the original strain.

The present analysis suggests that inhibitors designed to prevent binding to the host cell and thus virus infection should also consider negative charged entities that target the top domain of the S protein of SARS-CoV-2 VoCs and block its docking onto the HSPG. Indeed, a recent *in vivo* study showed the potential of a negatively charged polyacrylic acid-gelatin hydrogel that prevent SARS-CoV2 virus infections in monkeys (Mei et al., 2023). Chonira et al. have shown that a trimeric DARPin (Designed Ankyrin Repeat Protein) fused to a T4 fold-on bound strongly to the top domain of the S protein of SARS-CoV-2 variants (Chonira et al., 2023). The surface of DARPin is essentially of a negative potential as confirmed by an APBS analysis (not shown). The authors found that trimeric DARPin binds best to the Omicron variants (IC_50_: Wuhan–1834 pM; Delta (B.1.617.2)–183 pM; Omicron (B.1.1.529) – 7.3 pM). While specific residues of the RBM and DARPins are engaged, we deduce that electrostatic interactions are also involved in binding and are even more pronounced in the top domain of the Omicron variants due to the additional positively charged amino acids (Figures 3, 4).

### 3.3. SARS-CoV-1, MERS-CoV, and endemic human coronaviruses

Despite their homologous S proteins, human coronaviruses recognize different specific cellular receptors. Similar to the case of SARS-CoV-2, ACE2 serves as the host cell receptor for SARS-CoV-1 (Li et al., 2003), while the cognate receptor of MERS-CoV is dipeptidyl-aminopeptidase 4 (DPP4; Raj et al., 2013). Specific cellular receptors also vary among the endemic human coronaviruses HCoV-NL63, HCoV-OC43 and HCoV-229E. While HCoV-NL63 also utilizes ACE2 (Milewska et al., 2014), HCoV-OC43 specifically binds to acetyl neuraminic acid, while HCoV-229E uses aminopeptidase N for cell entry (Yeager et al., 1992). Neither the S protein of MERS-CoV nor those of HCoV-OC43, HCoV-NL63 and HCoV-229E contains a Cardin-Weintraub sequence (see www.uniprot.org entries K9N5Q8, A0A140E065, Q6Q1S2, P15432, respectively). Binding of monomeric S of SARS-CoV-1 but also of MERS-CoV to heparin was demonstrated by SPR, albeit binding is with much lower affinity in comparison to that of SARS-CoV-2 (Kim et al., 2020). However, MERS-CoV was shown to also employ sialic acid as a co-receptor along with its main receptor DPP4 (Li et al., 2017).

Recently, an inhibitory effect of lactoferrin on the infection of cell cultures by HCoV-OC43, HCoV-NL63, and HCoV-229E viruses was found. From the binding of lactoferrin to HS on the cell surface, it was concluded indirectly that HS may also serve as a co-receptor for these viruses (Hu et al., 2021). However, laboratory strains may have gained affinity to heparan sulfate due to mutations in the course of serial passages. For example, the potential furin cleavage site of the S protein of HCoV-OC43 -NRRSRRA- changed to a Cardin-Weintraub-motif -NRRSRGA- in the laboratory strain HCoV-OC43* (Figure 4C; de Haan et al., 2008). But direct evidence that HS acts as co-receptor of HCoV-OC43 and HCoV-229E on permissive host cells is missing. Milewska et al. (2014) have shown for HCoV-NL63 that ACE2 is required for cell entry but HSPG serve as a primary attachment molecule (Milewska et al., 2014). In a subsequent study it was found that the M membrane protein present in the HCoV-NL63 envelope but not S binds to HS (Naskalska et al., 2019). The 3D structure of this protein is unknown.

**Figure 6:**
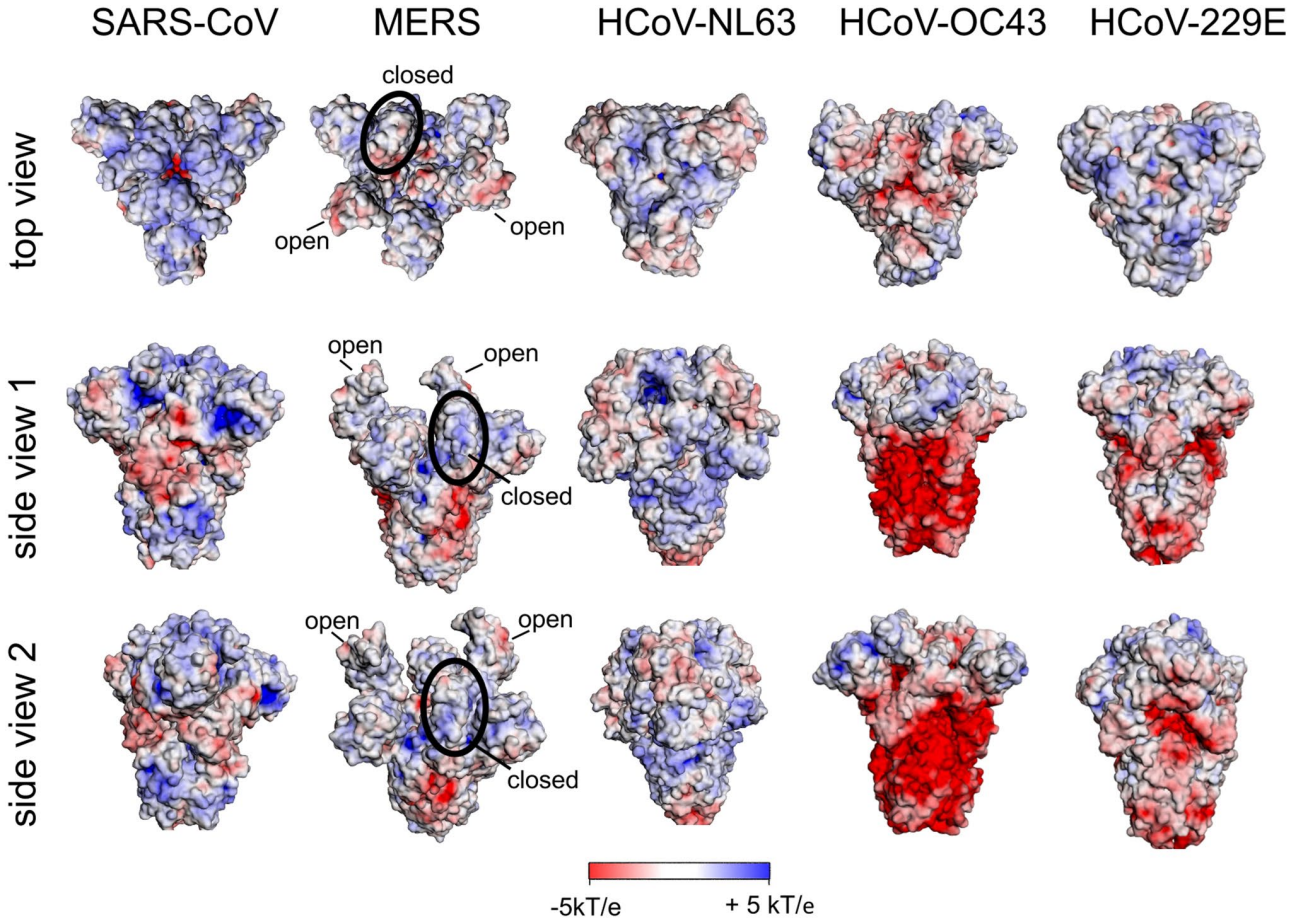
Surface potential of the ectodomain of the S protein of human coronaviruses. All structures are in the closed state of RBD except for MERS-CoV S. For the latter, two RBDs are in the open and one RBD in the closed state. A 3D structure with RBDs all in the closed state of RBD is not available [pdb: 5×58 (SARS-CoV); 5X5C (MERS-CoV); 5SZS (HCoV-NL63); 6OHW (pdb only available for HCoV-OC43*); 7CYC (HCoV-229E)].

We have calculated the surface potential of the S protein of SARS-CoV-1, MERS-CoV, HCoV-NL63, HCoV-OC43 and HCoV-229E (Figure 6) as described above. The top domain of the S protein of SARS-CoV-1 was comparable to that of the Wuhan SARS-CoV-2 rationalizing the observation that SARS-CoV-1 employs HS as a co-receptor (Lang et al., 2011). In contrast to the S protein of SARS-CoV-2, we could not identify domains of remarkable positive surface potential in the top domain or the stem region of S of MERS-CoV, HCoV-NL63, or HCoV-OC43. This finding is in accord with the fact that no Cardin-Weintraub sequences are found in the spike protein of these viruses (see above). Since HCoV-OC43 and MERS-CoV use the abundant neuraminic acid as a receptor and co-receptor (see above), respectively, we assume that naturally occurring variants of these viruses had no selective pressure or advantage to adapt to HS as a coreceptor. Based on the positive surface potential of the top domain of the HCoV-229E S protein, this could be a potential binding site for HS. However, up to now there is no evidence for this in the literature.

## 4. Human respiratory syncytial virus and human metapneumovirus

The relevance of a positive surface potential of the spike protein hemagglutinin of influenza A virus for interaction with the negatively charged host cell receptor sialic acid (Fantini et al., 2023b) and changes of the surface potential of hemagglutinin associated with the evolution and spreading of avian influenza A virus clades (Righetto and Filippini, 2020) have been discussed recently. Influenza A viruses (IAVs) use specific sialic acid residues (Sia) of the host cell glycocalyx as receptors. Thus, human pathogenic IAVs preferentially bind to α2,6-linked sialic acids, whereas avian IAVs preferentially bind to α2,3-linked sialic acids. The specificity might be less dichotomous that assumed before (Childs et al., 2009; Xu et al., 2012; Liu M. et al., 2022). For example, Liu M. et al. (2022) have concluded from studies on the binding behavior of an avian IAV that the binding specificity to sialic acids might be less dichotomous than previously assumed. Using a Sia-deficient HEK293 cell line, they systematically modified the composition of 2,3 and 2,6 sialoglycans on the cell surface by cotransfection with specific sialyltransferases. At the same time, they used sialoglycoproteins secreted by these cells to study binding of the virus by bilayer interferometry. The authors were able to show that binding and entry of the avian virus was significantly promoted by the human 2,6 Sia receptor when the avian 2,3 Sia receptor was only present to a small extent. Byrd-Leotis et al. (2019) found by binding to an N-glycan array that a range of avian, swine and human IAVs bind also to phosphorylated, nonsialylated N-glycans. Previous studies indicated the importance of the IAV hemagglutinin (HA) surface potential for virus binding to sialic acid residues on the host cell surface. Righetto and Filippini (2020) investigated the surface potential of the sialic acid binding domain of low pathogenic H5 IAV strains circulating and spreading among wild birds that could become highly pathogenic strains to poultry birds through genome variation. They found fingerprints of the binding domain surface potential specific for highly and low pathogenic H5 strains. In their review, Fantini et al. (2023a) pointed out that HA of IAV strains share the same arrangement of the cationic and aromatic residues as a canonical binding domain for sialic acids of negatively charged gangliosides (Suzuki, 1994), which is also used by other viruses such as Sendai virus (Markwell et al., 1981) and SV40 (Campanero-Rhodes et al., 2007) viruses [for more details see Fantini et al. (2023a) and references therein]. By analyzing the surface potential of the HA of the avian H5 IAV strain, Fantini et al. (2023a) hypothesized that the high positive surface potential of the HA tip of the ferret-transmissible H5 strain could allow the virus to infect multiple animal species and also become transmissible to humans. However, to our opinion, a more detailed analyses is still necessary to unravel the interplay between the stereo specific binding of HA to sialic acid residues of the host cell surface and the surface potential of the HA ectodomain for strain specific binding to host cells, and how a perturbation of this interaction is prevented by an interaction with HS.

Human respiratory syncytial virus (hRSV) and human metapneumovirus (hMPV) are important pathogens and cause infections in all age groups but predominantly affect infants. They preferentially infect the lower respiratory tract, mostly ciliated airway epithelial cells and type I alveolar pneumocytes (Schildgen et al., 2011; Battles and McLellan, 2019). Both viruses are members of the Pneumovirinae subfamily of the Paramyxoviridae family, and they are genetically closely related (Schildgen et al., 2011; Battles and McLellan, 2019). Two proteins are important for the early phases of virus infection are exposed on their envelope: the attachment protein (G) and the homotrimeric fusion protein (F). The F protein recognizes host cell-specific receptors and, after endocytic uptake of viruses, triggers the fusion of the viral envelope with the endosomal membrane of the host cell.

For both viruses it has been shown that the G protein binds to HS present in GAGs on the host cell surface (Chang et al., 2012; Klimyte et al., 2016) similar to the binding mechanism discussed above, namely, through a stretch of positively charged amino acids of a heparin-binding domain in the G protein. This domain is located between two mucin-like motifs as shown for hRSV (Krusat and Streckert, 1997; Feldman et al., 1999; Escribano-Romero et al., 2004). For both viruses, the G protein was found to be dispensable for virus replication *in vitro*, while the F protein is essential (Karron et al., 1997; Chang et al., 2012; Klimyte et al., 2016). It was shown that the F protein also mediates the initial binding of both viruses to the host cell through an interaction with cellular HS (Feldman et al., 2000; Chang et al., 2012). Based on the observations that both the G and F proteins of both viruses bind to HS, an analysis of the surface potential of the ectodomains of these proteins is of interest. However, this analysis is not possible for the G proteins because their ectodomains are disordered (Leyrat et al., 2014). One may hypothesize, however, that a flexible ectodomain enables an efficient interaction of positively charged amino acids with high content of negatively charged sialic acid and sulfate groups of mucus (Leal et al., 2017).

**Figure 7:**
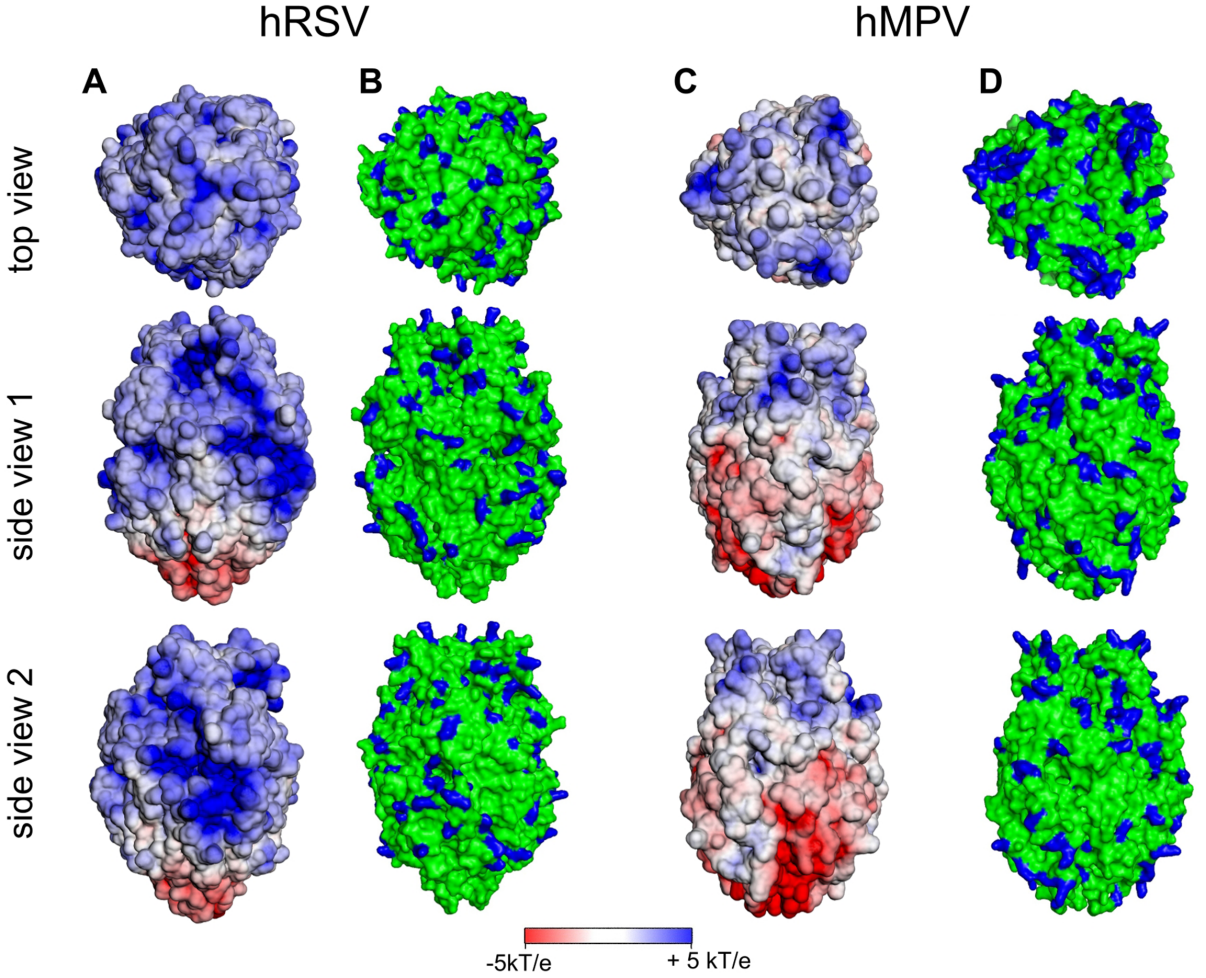
Surface potential **(A,C)** and surface distribution of positively charged amino acids Arg and Lys [blue; **(B,D)**] of human respiratory syncytial virus (hRSV; pdb 5UDE) and human metapneumovirus (hMPV; pdb 5WB0).

In contrast, the 3D structures of the ectodomains of the F proteins of both hRSV and hMPV are available. In Figure 7, we show the surface potential and the distribution of exposed and positively charged amino acids. As is obvious, both ectodomains are characterized by a pronounced positive surface potential that is more pronounced in the case of hRSV. Thus, binding to HS of the F proteins of either virus can be rationalized by the exposure of positively charged amino acids in the F ectodomains, which give rise to a positive surface potential as shown in Figure 7. Evidently, the analysis of the surface potential gives a more intuitive insight into the localization of positively charged patches than the surface distribution of positively charged amino acids. In consequence, this analysis strongly suggests for hRSV and hMPV a binding of the virion to the HSPG of the host cell as discussed above for SARS-CoV-2. The initial binding is mediated by interaction of G and F with HS. Subsequently, the F proteins recognize and interact with specific host cell receptors [for a review (Battles and McLellan, 2019)].

## 5. Conclusion

This survey has explored the relationship between positive surface charge patches on viral surface proteins with host cell binding. For SARS-CoV-2 this connection has clearly been shown: the first step of viral infection is the strong interaction of the positively charged patches on the surface of the spike protein with the negatively charged heparan sulfate chains on the cell surface. In a second step, cell entry is mediated by specific interaction with surface receptors such as ACE2. Surface potential maps generated by the Poisson-Boltzmann solver (APBS) were shown to provide a convenient tool to rationalize the interaction of the ectodomain of the virus with HS. A comparison with results for a number of different viruses underscored the validity of this approach. Hence, from our analysis and from data in literature, we may discern

(i)Viruses that need the interaction with HS for cell entry: SARS-CoV-2, Human respiratory syncytial virus (hRSV) and human metapneumovirus (hMPV)
(ii)Viruses that do not need this interaction: MERS, HCoV-NL63, and HCoV-OC43

The present analysis did not consider a possible change of the interaction by glycosylation of the spike proteins. This modification may affect the interaction considerably. However, the present survey suggests that glycosylation may alter the magnitude of binding constant but will not change the main conclusion presented here.

## Author contributions

DL, KO, RH, MB, and AH are contributed to conception. MB and AH design the study and performed the theory and the calculations, respectively. DL, MB, and AH wrote the first draft of the manuscript. All authors contributed to manuscript revision, read, and approved the submitted version.

## Funding

This work was funded by the Deutsche Forschungsgemeinschaft within the GRK 2662 “Charging into the Future” (DFG, German Research Foundation) Project ID 434130070 and the CRC 1449 “Dynamic Hydrogels at Biointerfaces” (DFG, German Research Foundation) Project ID 431232613 – SFB 1449.

## Conflict of interest

The authors declare that the research was conducted in the absence of any commercial or financial relationships that could be construed as a potential conflict of interest.

## Publisher’s note

All claims expressed in this article are solely those of the authors and do not necessarily represent those of their affiliated organizations, or those of the publisher, the editors and the reviewers. Any product that may be evaluated in this article, or claim that may be made by its manufacturer, is not guaranteed or endorsed by the publisher.
